# Fast algorithm and new potential formula represented by Chebyshev polynomials for an $m\times n$ globe network

**DOI:** 10.1038/s41598-022-25724-y

**Published:** 2022-12-08

**Authors:** Yufan Zhou, Yanpeng Zheng, Xiaoyu Jiang, Zhaolin Jiang

**Affiliations:** 1 School of Information Science and Engineering, Linyi University, Linyi, 276000 China; 2 School of Automation and Electrical Engineering, Linyi University, Linyi, 276000 China; 3 School of Mathematics and Statistics, Linyi University, Linyi, 276000 China

**Keywords:** Mathematics and computing, Physics

## Abstract

Resistor network is widely used. Many potential formulae of resistor networks have been solved accurately, but the scale of data is limited by manual calculation, and numerical simulation has become the trend of large-scale operation. This paper improves the general solution of potential formula for an $m\times n$ globe network. Chebyshev polynomials are introduced to represent new potential formula of a globe network. Compared with the original potential formula, it saves time to calculate the potential. In addition, an algorithm for computing potential by the famous second type of discrete cosine transform (DCT-II) is also proposed. It is the first time to be used for machine calculation. Moreover, it greatly increases the efficiency of computing potential. In the application of this new potential formula, the equivalent resistance formulae in special cases are given and displayed by three-dimensional dynamic view. The new potential formulae and the proposed fast algorithm realize large-scale operation for resistor networks.

## Introduction

With the development of natural science, people have encountered various new problems. According to the complexity of the problem, solutions emerge in endlessly. Researches show that a host of problems can be solved by establishing resistor network model^1–10^ and neural network model^11–17^. In the past few years, many results have been achieved in the study of resistor networks. For example, the establishment of graph theory, the researches on Laplacian matrix (*LM*) method of resistor network, infinite network, finite network, and corner-to-corner resistance and so on^7–10,18–31^. Shi^11,12^ et al. studied a novel discrete-time recurrent neural network. Liu^13^ et al. and Sun^14^ et al. proposed different types of the zeroing neural network. And Jin^15–17^ et al. studied an innovative control theory stimulated gradient neural network algorithm.

In recent years, Tan^32–43^ proposed the Recursion-Transform (*RT*) method which is different from the Laplacian matrix method. It only depends on one matrix and one direction, and the calculation is simpler. In 2013, Tan^32^ studied the problem of two-point resistance on cobweb with a 2*r* boundary, which has never been solved before. Under finite and infinite conditions, the $m \times n$ cobweb general formula of resistance between any two nodes is proposed. In 2014, Tan^36,38^ made a new breakthrough and introduced the equivalent resistance of the globe network and fan network model by *RT* method. Shortly after that many researches on resistor networks are based on *RT* method. *RT* method needs eigenvalues of a tridiagonal matrix to represent the potential formula. At present, there have been many results on tridiagonal matrices^45–51^, which are also widely used. It can be said that it is a powerful tool to solve the resistor network^33–43^.

**Figure 1:**
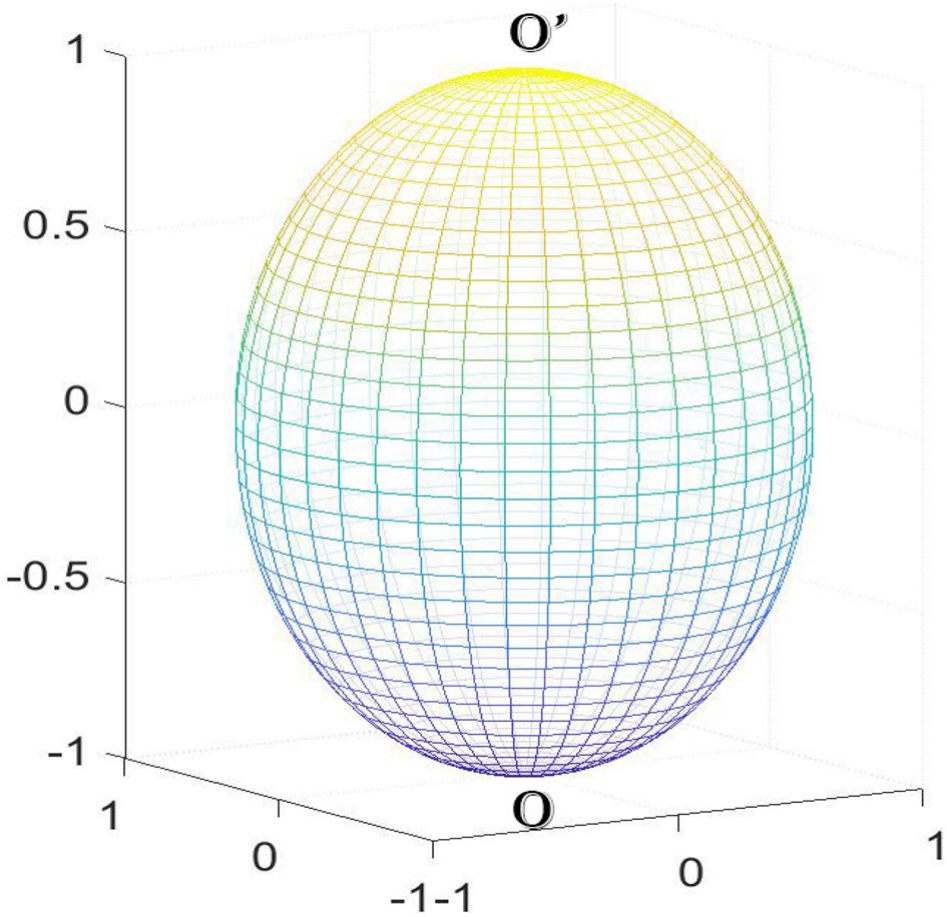
An *m* $\times$ *n* globe network which has *n* longitude and $m-1$ latitude. The resistances in the longitude and latitude directions are $r_{0}$ and *r*, respectively.

**Figure 2:**
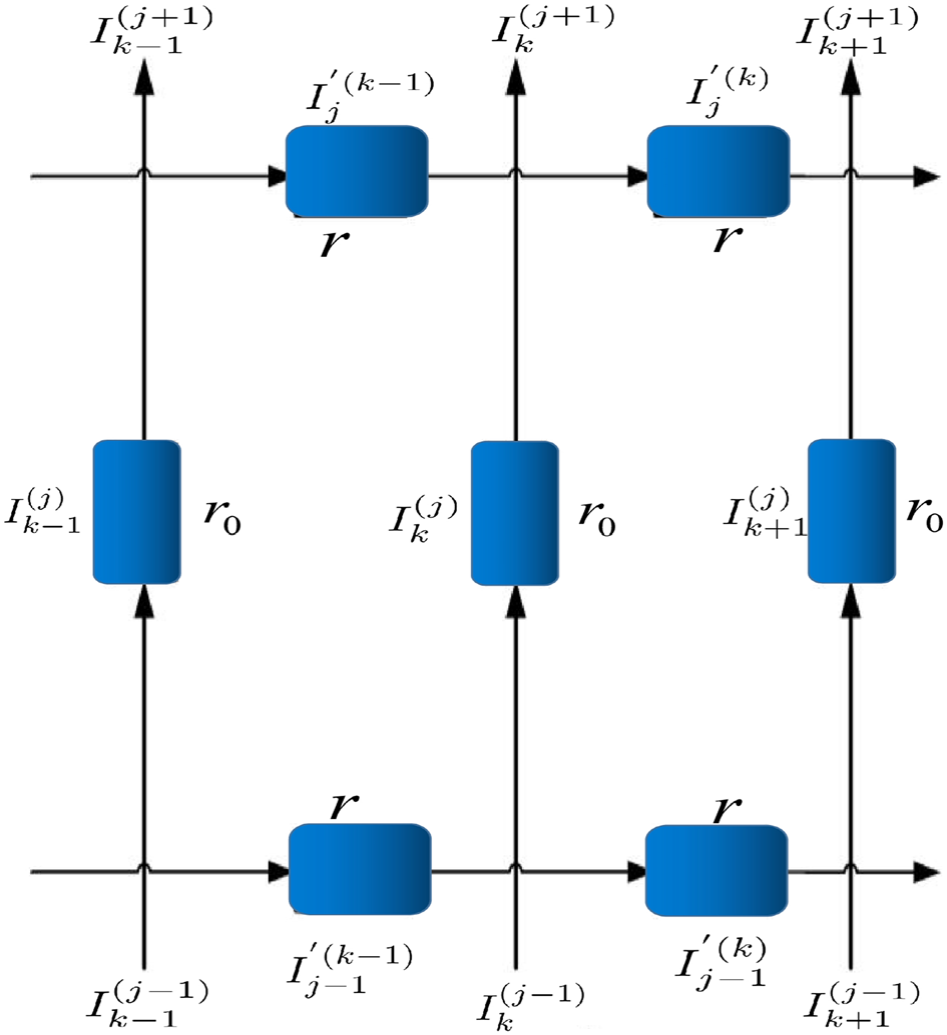
Segment of the globe network with current directions and parameters.

In 2018, Tan^44^ proposed an $m \times n$ globe network, as shown in Fig. 1. The resistances in the latitude and longitude directions are *r* and $r_{0}$, respectively, where *m* and *n* are the resistance numbers along the latitude and longitude directions. The nodes of the network are represented by coordinates $\{x, y\}$. Considering $O_{0}=0$ as the origin of the coordinate system, the potential of node *d*(*x*, *y*) is $U_{m\times n}(x , y )$ as shown in Fig. 2. The potential of any node in the $m\times n$ globe network is as follows

1 $$\begin{aligned} \frac{U_{m\times n}(x,y)}{J} = \frac{y(y_{1}-y_{2})}{mn}r_{0} + \frac{2r}{m}\sum ^{m}_{i=2}\frac{\beta ^{(i)}_{x_{1},x}S_{y_{1},i}-\beta ^{(i)}_{x_{2},x}S_{y_{2},i}}{\lambda ^{n}_{i}+\bar{\lambda }^{n}_{i}-2}S_{y,i}, \end{aligned}$$

where

2 $$\begin{aligned} S_{k,i}= & \sin (y_{k}\theta _{i}),\ \theta _{i} = (i-1)\pi /m, \end{aligned}$$

3 $$\begin{aligned} \beta ^{(i)}_{x_{s},x_{k}}= & F_{n-|x_{s}-x_{k}|}+F_{|x_{s}-x_{k}|},\ F^{(i)}_{k} = (\lambda ^{k}_{i}-\bar{\lambda }^{k}_{i})/(\lambda _{i}-\bar{\lambda }_{i}), \end{aligned}$$

4 $$\begin{aligned} \lambda _{i}= & 1+b-b\cos \theta _{i}+\sqrt{(1+b-b\cos \theta _{i})^{2} - 1},\nonumber \\ \bar{\lambda }_{i}= & 1+b-b\cos \theta _{i}-\sqrt{(1+b-b\cos \theta _{i})^{2} - 1}, ~~b=r/r_{0}. \end{aligned}$$

## New formula of potential represented by Chebyshev polynomials

Since the potential formula (1) contains exponential function operation, it is less efficient to calculate potential of globe resistor networks by computer. The purpose of this section is to give an improved potential formula of the globe network^44^, i.e. a new formula of potential expressed by Chebyshev polynomial of the second class is given^53^. it saves time to calculate the potential by computer.

Let the current *J* from $d_{1}(x_{1}, y_{1})$ to $d_{2}(x_{2}, y_{2})$, the potential formula of two nodes in an $m \times n$ resistor network can be written as

5 $$\begin{aligned} \frac{U_{m\times n}(x,y)}{J} = \frac{y(y_{1}-y_{2})}{mn}r_{0} + \frac{2r}{m}\sum ^{m}_{j=2}\frac{\gamma ^{(j)}_{x_{1},x}C_{y_{1},j}-\gamma ^{(j)}_{x_{2},x}C_{y_{2},j}}{t_{j}U^{(j)}_{n-1}-2U^{(j)}_{n-2}-2}C_{y,j}, \end{aligned}$$

where

6 $$\begin{aligned} & \gamma ^{(j)}_{x_{s},x_{k}} = U^{(j)}_{n-|x_{s}-x_{k}|-1}+U^{(j)}_{|x_{s}-x_{k}|-1}, \ s = 1,2, \end{aligned}$$

7 $$\begin{aligned} & \quad C_{p,j} = \sin (\frac{p(j-1)\pi }{m}),\ p=y_{1},\ y_{2},\ y, \end{aligned}$$

8 $$\begin{aligned} & \quad t_{j}=2+\frac{2r}{r_{0}}-\frac{2r}{r_{0}}\cos \frac{(j-1)\pi }{m}, \end{aligned}$$

9 $$\begin{aligned} & \quad U^{(j)}_{k} = U^{(j)}_{k}(\cos \psi _{j}) = \frac{\sin (k+1)\psi _{j}}{\sin (\psi _{j})},\ \psi _{j}=\arccos (1+\frac{r}{r_0}-\frac{r}{r_0}\cos \frac{(j-1)\pi }{m}),\nonumber \\ & \quad k=n-|x_1-x|,~|x_1-x|,~n-|x_2-x|,~|x_2-x|,~n-1,~n-2,~~j=1,2,\ldots , m. \end{aligned}$$

In particular, the input and output points can be one or more, so formula (5) applies to all coordinate points $(x_{k}, y_{k})$ $(0\le k \le n)$. Therefore, *p* can also be $y_3,\ y_4,\ldots ,\ y_k$.

Suppose that $O_{0} = 0$, then calculating the node potential between any two points by Ohm’s law can be described as

10 $$\begin{aligned} U_{m\times n}(x,y) = U(0,0) - r_{0}\sum ^{y}_{j=1}I^{(j)}_{x} = -r_{0}\sum ^{y}_{j=1}I^{(j)}_{x}, \end{aligned}$$

where $I_{x}^{(j)}$ is denoted by the current in the vertical direction.

**Figure 3:**
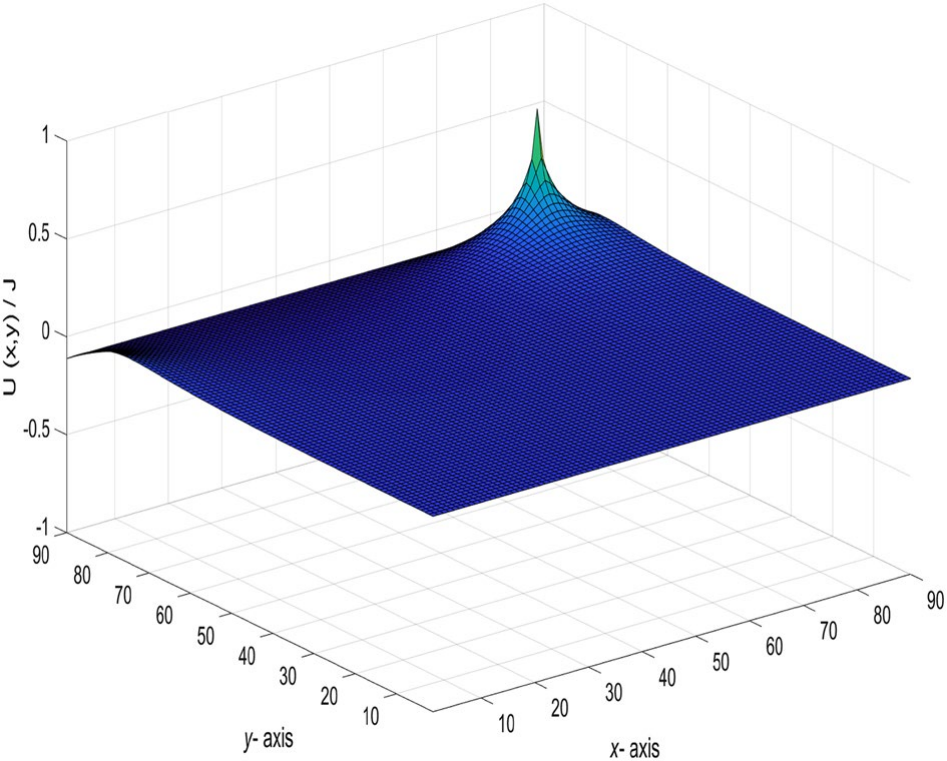
A 3D dynamic view for the changing graph of $U_{90\times 90}(x,y)/J$ with the current input and output point change.

Base on formula (5), using the visualization of data to realize the dynamic visualization of the potential change. With the change of the current input and output point change, the potential change of any two nodes is shown in Fig. 3.

## Three terms recurrence sequence and discrete cosine transform

In this section, in order to improve the actual performance and realize the fast algorithm, the three terms recurrence sequence represented by Chebyshev polynomial of the second class and two types of discrete cosine transform are introduced.

The three terms recurrence sequence is defined by the following conditions:

11 $$\begin{aligned} W_k=dW_{k-1}-qW_{k-2},~~W_0=A,~~W_1=B, \end{aligned}$$

where $k\in \textbf{N},~~k\ge 2,~~A,B,d,q\in \textbf{C}$, $\textbf{N}$ is the set of all natural numbers and $\textbf{C}$ is the set of all complex numbers.

The three terms recurrence sequence^52^ represented by Chebyshev polynomial of the second class is

12 $$\begin{aligned} W_{k} =(\sqrt{q})^k\left( \frac{B}{\sqrt{q}}U_{k-1}\left( \frac{d}{2\sqrt{q}}\right) - AU_{k-2}\left( \frac{d}{2\sqrt{q}}\right) \right) , \end{aligned}$$

where

13 $$\begin{aligned} U_{k} = \frac{\sin (k+1)\psi }{\sin \psi },~~\psi =\arccos (\frac{d}{2\sqrt{q}})\in \textbf{C}, \end{aligned}$$

is the Chebyshev polynomial of the second class^53^.

### Remark

Based on the above formula, how to replace Eqs. (1), (2), (3) and (4) with Eqs. (5), (6), (7) and (9) will be showed, respectively. According to the equation (4), $\lambda _{j}+\bar{\lambda }_{j}=t_{j}$ and $\lambda _{j}\cdot \bar{\lambda }_{j}=1$ are obtained . Take it into the equation (11), and a three term recursive formula is obtained

14 $$\begin{aligned} F^{(j)}_k=t_{j}F^{(j)}_{k-1}-F^{(j)}_{k-2},~~F^{(j)}_{0}=0,~~F^{(j)}_{1}=1, \end{aligned}$$

where $d=t_{j},\ q=1$, $t_{j}$ and $F^{(j)}_{k}$ are the same as Eqs. (8) and (3), respectively. By Eq. (14) and formula (12), the replacement result can be obtained

15 $$\begin{aligned} {F^{(j)}_{k} = (\lambda ^{k}_{j}-\bar{\lambda }^{k}_{j})/(\lambda _{j}-\bar{\lambda }_{j})=U^{(j)}_{k-1}(\frac{t_{j}}{2}).} \end{aligned}$$

Similarly, Let

16 $${Q^{(j)}_{n}=\lambda ^{n}_{j}+\bar{\lambda }^{n}_{j},}$$

where $Q^{(j)}_{0}=2,\ Q^{(j)}_{1}=t_{j}$. Then the recursive relation of $Q^{(j)}_{n}$ is obtained as follows

17 $$\begin{aligned} {Q^{(j)}_{n}=t_{j}Q^{(j)}_{n-1}-Q^{(j)}_{n-2},\ Q^{(j)}_{0}=2,\ Q^{(j)}_{1}=t_{j},} \end{aligned}$$

where $d=t_{j},\ q=1$, $t_{j}$ and $Q^{(j)}_{n}$ are the same as Eqs. (8) and (16), respectively. By Eq. (17) and formula (12), the replacement result can be obtained as follows

18 $$\begin{aligned} {Q^{(j)}_{n}=\lambda ^{n}_{j}+\bar{\lambda }^{n}_{j}=t_{j}U_{n-1}(\frac{t_{j}}{2})-U_{n-2}(\frac{t_{j}}{2}).} \end{aligned}$$

According to Eqs. (1), (3),(15) and (18), a new potential formula (5) is got.

In order to obtain a fast numerical algorithm for computing the potential, the orthogonal diagonalization of the matrix $\textbf{B}_m$ is given.

19 $$\begin{aligned} \textbf{B}_{m}= \left( \begin{array}{cccccc} 2+b & -b & 0& \cdots & 0\\ -b & 2(1+b) & -b & \ddots & \vdots \\ 0 & \ddots & \ddots & \ddots & 0 \\ \vdots & \ddots & -b & 2(1+b) & -b\\ 0 & \cdots & 0 & -b & 2+b \end{array} \right) _{m\times m}, \end{aligned}$$

where $$b = r/r_0$$.

Then the eigenvalues $t_1,\ldots ,t_m$ of $\textbf{B}_{m}$ are given by

20 $$\begin{aligned} t_j=2+2b-2b\cos \frac{(j-1)\pi }{m},~~j=1,2,\ldots ,m, \end{aligned}$$

and the corresponding eigenvectors $\zeta ^{(j)}=(\zeta ^{(j)}_1,\ldots ,\zeta ^{(j)}_m)^\dag$ are given by

21 $$\begin{aligned} \zeta ^{(j)}_k=\sqrt{\frac{2}{m}}d_j\cos \frac{(2k-1)(j-1)\pi }{2m},~~k=1,2,\ldots ,m,j=1,2,\ldots , m, \end{aligned}$$

where $$d_j={\left\{ \begin{array}{ll}1,&j\ne 1,\\ \frac{\sqrt{2}}{2},&j=1.\end{array}\right. }$$

Let

22 $$\begin{aligned} \mathbb {C}_m^{III}=\left( \sqrt{\frac{2}{m}}d_j\cos \frac{(2k-1)(j-1)\pi }{2m} \right) _{k,j=1}^m, \end{aligned}$$

where $$d_j={\left\{ \begin{array}{ll}1,&j\ne 1,\\ \frac{\sqrt{2}}{2},&j=1.\end{array}\right. }$$

Obviously, the matrix $\mathbb {C}_m^{III}$ is the famous third type of discrete cosine transform (DCT-III)^54–57^. $\mathbb {C}_m^{III}$ is an orthogonal matrix and the inverse of $\mathbb {C}_m^{III}$ is actually $\mathbb {C}_m^{II}$, i.e.

23 $$\begin{aligned} (\mathbb {C}_m^{III})^{-1}=(\mathbb {C}_m^{III})^T=\mathbb {C}_m^{II}, \end{aligned}$$

where the orthogonal matrix $\mathbb {C}_m^{II}$ is the famous second type of discrete cosine transform (DCT-II)^54–57^.

By calculation we obtain the orthogonal diagonalization of the matrix $\textbf{B}_{m}$ as follows

24 $$\begin{aligned} (\mathbb {C}_m^{III})^{-1}\textbf{B}_{m}(\mathbb {C}_m^{III})=\textrm{diag}(t_1,t_2,\ldots ,t_m), \end{aligned}$$

i.e.

25 $$\begin{aligned} \textbf{B}_{m}=(\mathbb {C}_m^{III})\textrm{diag}(t_1,t_2,\ldots ,t_m)(\mathbb {C}_m^{III})^{-1}, \end{aligned}$$

where $t_j, j=1,2,\ldots , m$ are given by Eq. (20).

Tan^44^ used Kirchhoff’s law to analyze the resistor network and to establish the resistor network model. The following general matrix equation is

26 $$\begin{aligned} \textbf{I}_{k+1} = \textbf{B}_{m}\textbf{I}_{k} - \textbf{I}_{k-1} - b \textbf{H}_{y}\delta _{k,x}, \end{aligned}$$

where $\textbf{B}_{m}$ is given by Eq. (19), the function $\delta _{k,x}$ is defined as: $\delta _{k,x}(x =k) = 1$, $\delta _{k,x}(x\ne k) = 0$, $\textbf{I}_{k}$ and $\textbf{H}_{x}$ are the $m \times 1$ column matrices, and can be written as

27 $$\begin{aligned} & \textbf{I}_{k} = [I^{(1)}_{k},I^{(2)}_{k},...I^{(m)}_{k}] ^{T}\ (0 \le k \le n), \end{aligned}$$

28 $$\begin{aligned} & \quad (\textbf{H}_{1})_{k} = J(-\delta _{k,y_{1}}+\delta _{k,y_{1}+1}),~~ (\textbf{H}_{2})_{k} = J(\delta _{k,y_{2}}-\delta _{k,y_{2}+1}). \end{aligned}$$

To solve the Eq. (26), using the matrix transformation method to multiply $\mathbb {C}_m^{III}$ at the same time. Through Eq. (24) and the transformed matrix equation we appoint

29 $$\begin{aligned} \sqrt{\frac{m}{2}}\mathbb {C}_m^{III}\textbf{I}_{k} = \textbf{W}_{k}\ and\ \textbf{I}_{k} = \sqrt{\frac{2}{m}}(\mathbb {C}_m^{III})^T\textbf{W}_{k} =\sqrt{\frac{2}{m}}\mathbb {C}_m^{II}\textbf{W}_{k}, \end{aligned}$$

where the $m\times 1$ column matrix $\textbf{W}_{k}$ is

30 $$\begin{aligned} \textbf{W}_{k} = [W^{(1)}_{k},W^{(2)}_{k},...,W^{(m)}_{k}]^{T} (0 \le k \le n). \end{aligned}$$

Based on the Chebyshev polynomial of the second class, the general solution formula of current is rewrited . When $j = 1$, the following formula is obtained

31 $$\begin{aligned} W^{(1)}_{k} = \frac{J(y_{2}-y_{1})}{n\sqrt{2}},(0\le k\le n). \end{aligned}$$

When $j\ge 2$, the following formula is obtained

32 $$\begin{aligned} W^{(j)}_{x} = bJ\frac{\gamma ^{(j)}_{x_{1},x}\varsigma _{1,j}+\gamma ^{(j)}_{x_{2},x}\varsigma _{2,j}}{t_{j}U^{(j)}_{n-1}-2U^{(j)}_{n-2}-2},(0\le x\le n), \end{aligned}$$

where $\gamma ^{(j)}_{x_{s},x_{k}}$ is equal to Eq. (6), $\varsigma _{1,j}= -2\sin (\frac{(j-1)\pi }{2m})\sin (\frac{y_{1}(j-1)\pi }{m})$, and $\varsigma _{2,j}= 2\sin (\frac{(j-1)\pi }{2m})\sin (\frac{y_{2}(j-1)\pi }{m})$.

## Displaying of some special and interesting potential formulae

Since formula (5) is a general potential conclusion of a globe network including all cases, some special conditions in formula (5) and a series of fascinating results under various parameters will be displayed. In the following, assuming that the potential reference at point *O*(0, 0) is $U(0, 0) = 0$.

### Special 1

Suppose the current *J* flows in from the origin $d_{1}(x_{1}, y_{1})$ and out from $d_{2}(x_{2}, y_{2})=O(0, 0)$, the potential of any two points can be written as

33 $$\begin{aligned} \frac{U_{m\times n}(x,y)}{J} = \frac{yy_{1}}{mn}r_{0} + \frac{2r}{m}\sum ^{m}_{j=2}\left( \frac{\sin (\frac{y_{1}(j-1)\pi }{m}))\sin (\frac{y(j-1)\pi }{m})}{t_{j}U^{(j)}_{n-1}-2U^{(j)}_{n-2}-2}\right) \gamma ^{(j)}_{x_{1},x}, \end{aligned}$$

where $\gamma ^{(j)}_{x_{s},x_{k}} = U^{(j)}_{n-|x_{s}-x_{k}|-1}+U^{(j)}_{|x_{s}-x_{k}|-1}$ is defined in Eq. (6), and $U^{(j)}_{k}$ is the same as Eq. (9).

When $m = n = 90,\ J = 10,\ x_{1} = y_{1} = 50,\ x_{2} = y_{2} = 0,\ and \ r_{0}=r=1,$ the following formula is obtained

34 $$\begin{aligned} \frac{U_{90\times 90}(x,y)}{J} = \frac{y}{162} + \frac{1}{45}\sum ^{90}_{j=2}\left( \frac{\sin (\frac{5(j-1)\pi }{9})\sin (\frac{(j-1)\pi }{90})}{t_{j}U^{(j)}_{89}-2U^{(j)}_{88}-2}\right) \gamma ^{(j)}_{50,x}, \end{aligned}$$

where

35 $$\begin{aligned} t_{j}&= 4 - 2\cos \frac{(j-1)\pi }{90}, \end{aligned}$$

36 $$\begin{aligned} \gamma ^{(j)}_{50,x}&= U^{(j)}_{89-|50-x|}+U^{(j)}_{|50-x|-1}, \end{aligned}$$

37 $$\begin{aligned} U^{(j)}_{k}&= U^{(j)}_{k}(\cos \psi _{j}) = \frac{\sin (k+1)\psi _{j}}{\sin (\psi _{j})},\ \psi _{j}=\arccos (2 - \cos \frac{(j-1)\pi }{90}),\nonumber \\ k&= 89-|50-x|,~|50-x|-1,~89,~88,~~j=1,2,\ldots ,90. \end{aligned}$$

**Figure 4:**
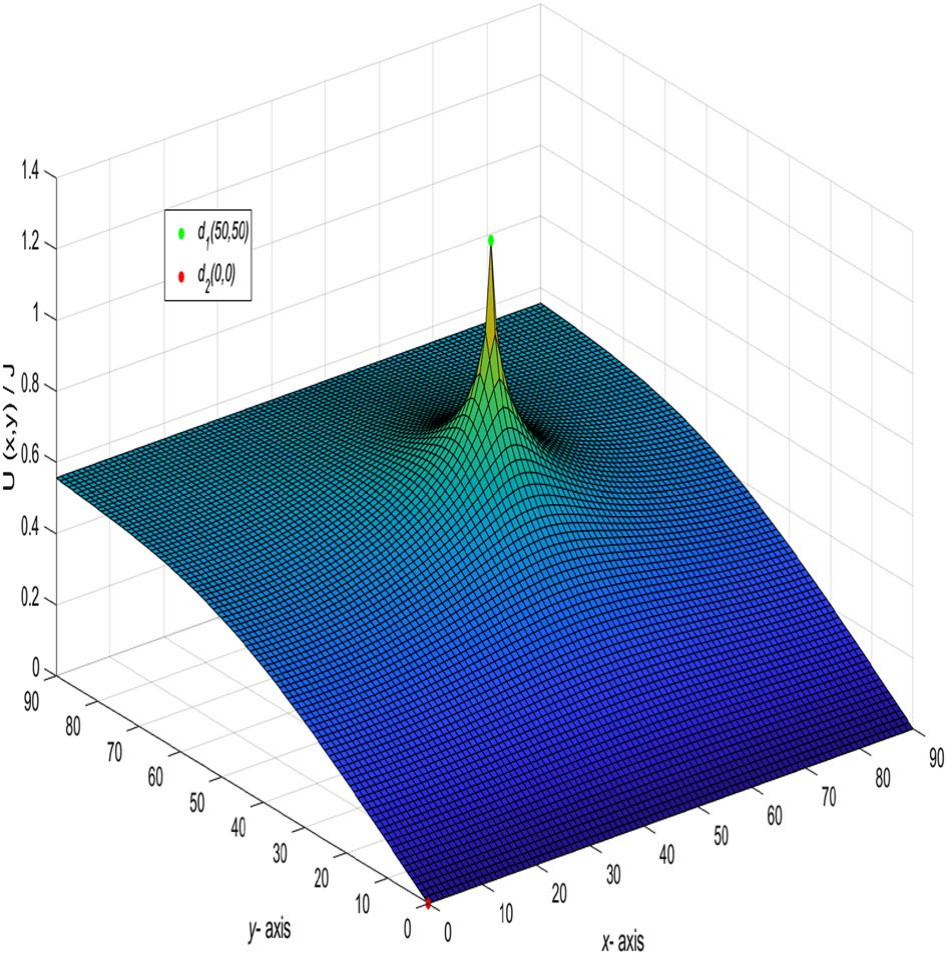
The process of forming a 3D dynamic view for $U_{90\times 90}(x,y)/J$ in Eq. (34).

And a 3D dynamic view is shown in Fig. 4 by Matlab.

**Figure 5:**
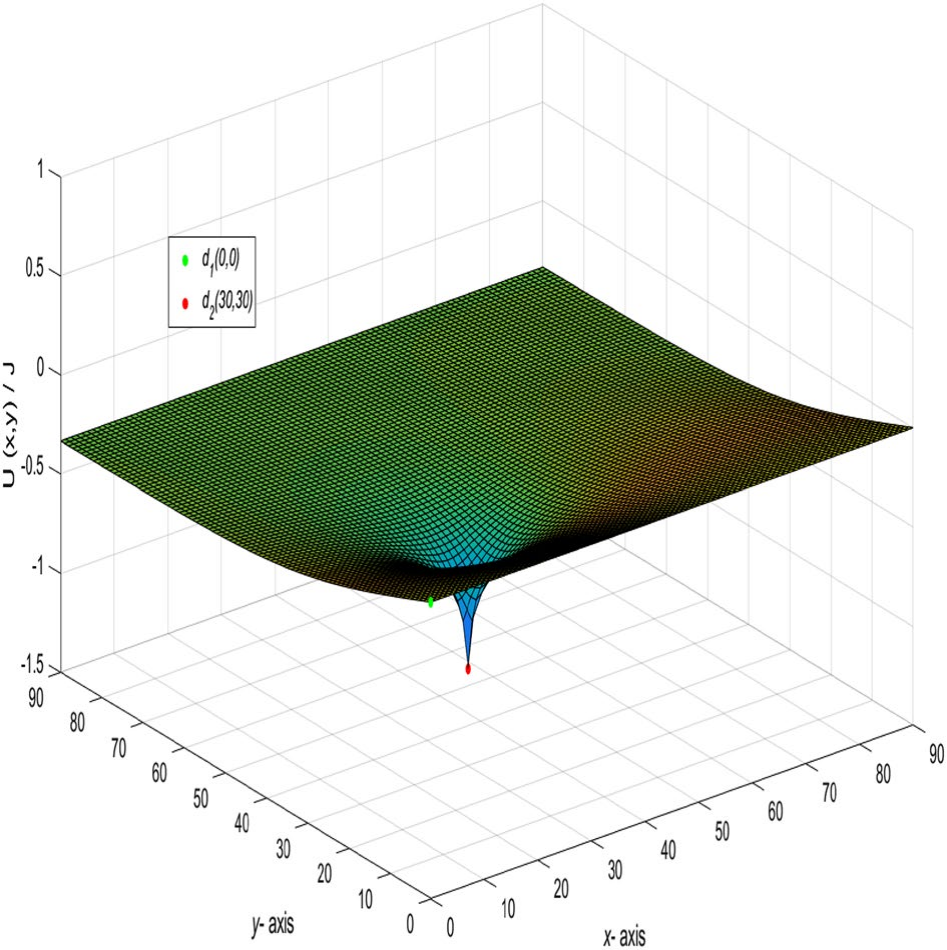
The process of forming a 3D dynamic view for $U_{90\times 90}(x,y)/J$ in Eq. (39).

### Special 2

Consider an arbitrary $m \times n$ globe network as shown in Fig. (1). Assume the electric current *J* outflow the network from the pole $d_{2}(x_{2}, y_{2})$, and the current *J* input at the node $d_{1}(x_{1}, y_{1})=O(0, 0)$, the potential of an arbitrary node *d*(*x*, *y*) can be written as

38 $$\begin{aligned} \frac{U_{m\times n}(x,y)}{J} = -\frac{yy_{2}}{mn}r_{0} + \frac{2r}{m}\sum ^{m}_{j=2}\left( -\frac{\sin (\frac{y_{2}(j-1)\pi }{m})\sin (\frac{y(j-1)\pi }{m})}{t_{j}U^{(j)}_{n-1}-2U^{(j)}_{n-2}-2}\right) \gamma ^{(j)}_{x_{2},x}, \end{aligned}$$

where $\gamma ^{(j)}_{x_{s},x_{k}}$ is defined in Eq. (6), and $U^{(j)}_{k}$ is the same as Eq. (9).

In the network with $m = 90$ and $n = 90$, the current flows in from $(x_{1},y_{1})(x_{1} =0,y_{1} = 0)$ and out from $(x_{2},y_{2})(x_{2} =30,y_{2} = 30)$. Let $r_{0} = r = 1$ and $J = 10$, the formula is deduced.

39 $$\begin{aligned} \frac{U_{90\times 90}(x,y)}{J} = -\frac{y}{270} + \frac{1}{45}\sum ^{90}_{j=2}\left( \frac{-\sin (\frac{(j-1)\pi }{3})\sin (\frac{(j-1)\pi }{90})}{t_{j}U^{(j)}_{89}-2U^{(j)}_{88}-2}\right) \gamma ^{(j)}_{30,x}, \end{aligned}$$

where

40 $$\begin{aligned} \gamma ^{(j)}_{30,x} = U^{(j)}_{89-|30-x|}+U^{(j)}_{|30-x|-1}, \end{aligned}$$

$t_{j}$ and $U^{(j)}_{k}$ are the same as Eqs. (35) and (37), respectively, $k=89-|30-x|,~|30-x|-1,~89,~88,~~j=1,2,\ldots ,90.$ And a 3D dynamic view is shown in Fig. 5 by Matlab.

### Special 3

Presume that when the current *J* is injected at node $d_{1}(x_{1}, y_{1})$ and withdrawn at node $d_{2}(x_{2}, y_{2})(x_{2}=y_{1},y_{2}=x_{1})$, the potential of any node *d*(*x*, *y*) is

41 $$\begin{aligned} \frac{U_{m\times n}(x,y)}{J} = \frac{yy_{1}}{mn}r_{0} + \frac{2r}{m}\sum ^{m}_{j=2}\frac{(U^{(j)}_{n-x}+U^{(j)}_{x})\sin (\frac{y_{1}(j-1)\pi }{m})\sin (\frac{y(j-1)\pi }{m})}{t_{j}U^{(j)}_{n-1}-2U^{(j)}_{n-2}-2}, \end{aligned}$$

where $t_{j}$ is defined in Eq. (8), and $U^{(j)}_{k}$ is the same as Eq. (9).

In the scale of $m \times n (90\times 90)$, we get $J = 10,\ x_{1} = y_{2}= 0,\ x_{2}=y_{1} = 30, \ and \ r_{0}=r=1,$ the following formula is

42 $$\begin{aligned} \frac{U_{90\times 90}(x,y)}{J} = \frac{y}{270} + \frac{1}{45}\sum ^{90}_{j=2}\frac{(U^{(j)}_{90-x}+U^{(j)}_{x})\sin (\frac{(j-1)\pi }{3})\sin (\frac{y(j-1)\pi }{90})}{t_{j}U^{(j)}_{89}-2U^{(j)}_{88}-2}, \end{aligned}$$

where $t_{j}$ and $U^{(j)}_{k}$ are the same as Eq. (35) and Eq. (37), respectively, and $k=90-x,~x,~89,~88,~~j=1,2,\ldots ,90.$

**Figure 6:**
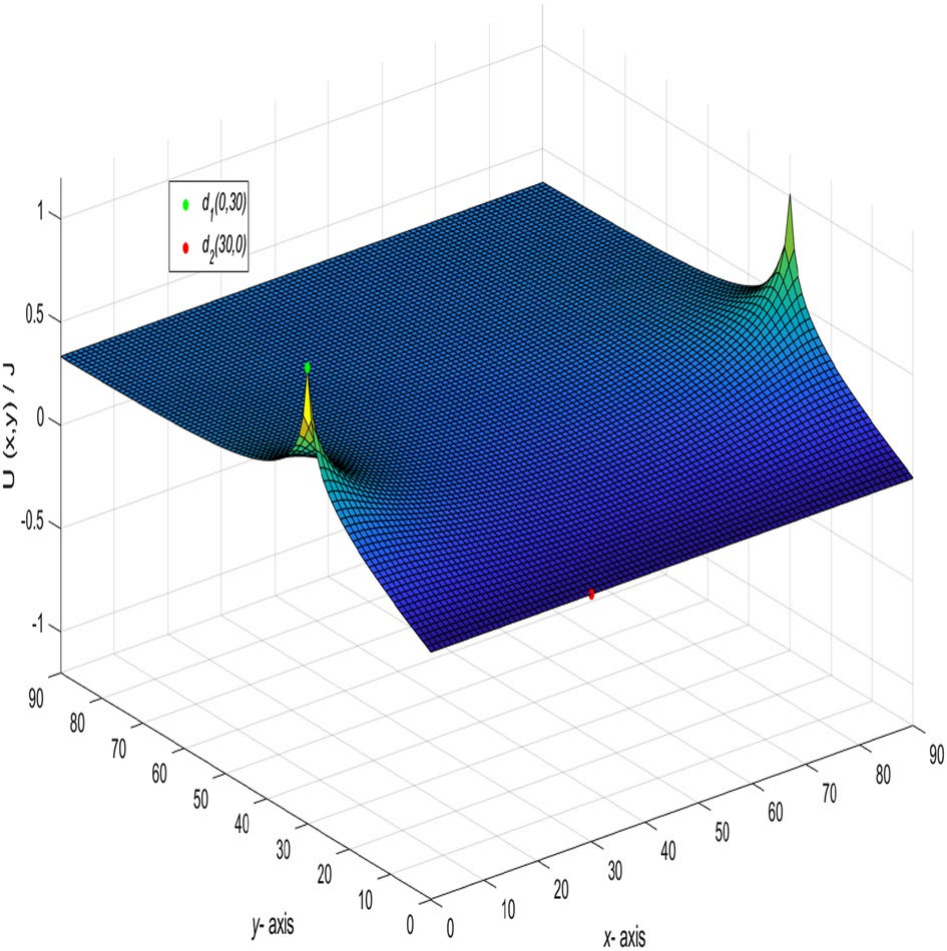
The process of forming a 3D dynamic view for $U_{90\times 90}(x,y)/J$ in Eq. (42).

And a 3D dynamic view is shown in Fig. 6 by Matlab.

**Figure 7:**
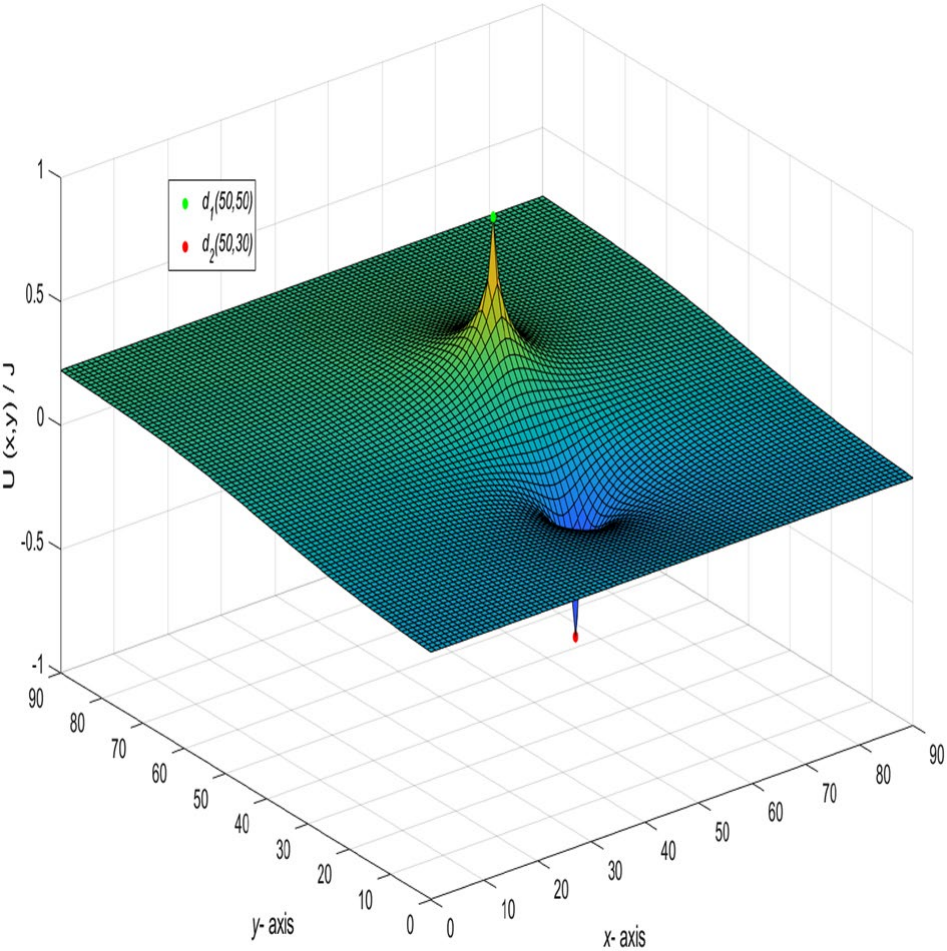
The process of forming a 3D dynamic view for $U_{90\times 90}(x,y)/J$ in Eq. (44).

### Special 4

When we inject current *J* at node $d_{1}(x_{1}, y_{1})$ and exit the current *J* at node $d_{2}(x_{1}, y_{2})(x_{2} = x_{1})$, the potential of an arbitrary node *d*(*x*, *y*) is

43 $$\begin{aligned} \frac{U_{m\times n}(x,y)}{J} = \frac{y(y_{1}-y_{2})}{mn}r_{0} + \frac{2r}{m}\sum ^{m}_{j=2}\left( \frac{(C_{y_{1},j} - C_{y_{2},j})C_{y,j}}{t_{j}U^{(j)}_{n-1}-2U^{(j)}_{n-2}-2}\right) \gamma ^{(j)}_{x_{1},x}, \end{aligned}$$

where $C_{k,j}$, $U^{(j)}_{k}$ and $\gamma ^{(j)}_{x_{1},x}$ are equal to Eqs. (7), (9) and Eq. (6), respectively.

When $m = n = 90,\ J = 10,\ x_{1} = x_{2} = y_{1} = 50,\ y_{2} = 30,\ and \ r_{0}=r=1,$ the following formula is obtained

44 $$\begin{aligned} \frac{U_{90\times 90}(x,y)}{J} = \frac{y}{405} + \frac{1}{45}\sum ^{90}_{j=2}\left( \frac{(C_{50,j} - C_{30,j})C_{y,j}}{t_{j}U^{(j)}_{89}-2U^{(j)}_{88}-2}\right) \gamma ^{(j)}_{50,x}, \end{aligned}$$

where

45 $$\begin{aligned} C_{50,j}= & \sin (\frac{5(j-1)\pi }{9}),\nonumber \\ C_{30,j}= & \sin (\frac{(j-1)\pi }{3}), \end{aligned}$$

$t_{j}$, $U^{(j)}_{k}$ and $\gamma ^{(j)}_{50,x}$ are the same as Eq. (35), Eq. (37)($k=~89,~88,~~j=1,2,\ldots ,90$) and Eq. (36), respectively.

And a 3D dynamic view is shown in Fig. 7 by Matlab.

### Special 5

When the current *J* enters from node $d_{1}(x_{1}, y_{1})$ to node $d_{2}(x_{2}, y_{1})$, in other words, $y_{1} = y_{2}$. From Eq. (5), the potential of any node *d*(*x*, *y*) can be written as

46 $$\begin{aligned} \frac{U_{m\times n}(x,y)}{J} = \frac{2r}{m}\sum ^{m}_{j=2}\left( \frac{\gamma ^{(j)}_{x_{1},x} - \gamma ^{(j)}_{x_{2},x}}{t_{j}U^{(j)}_{n-1}-2U^{(j)}_{n-2}-2}\right) C_{y_{1},j}C_{y,j}, \end{aligned}$$

where $\gamma ^{(j)}_{x_{s},x_{k}}$, $U^{(j)}_{k}$ and $C_{k,j}$ are equal to Eq. (6), Eq. (9) and Eq. (7), respectively.

**Figure 8:**
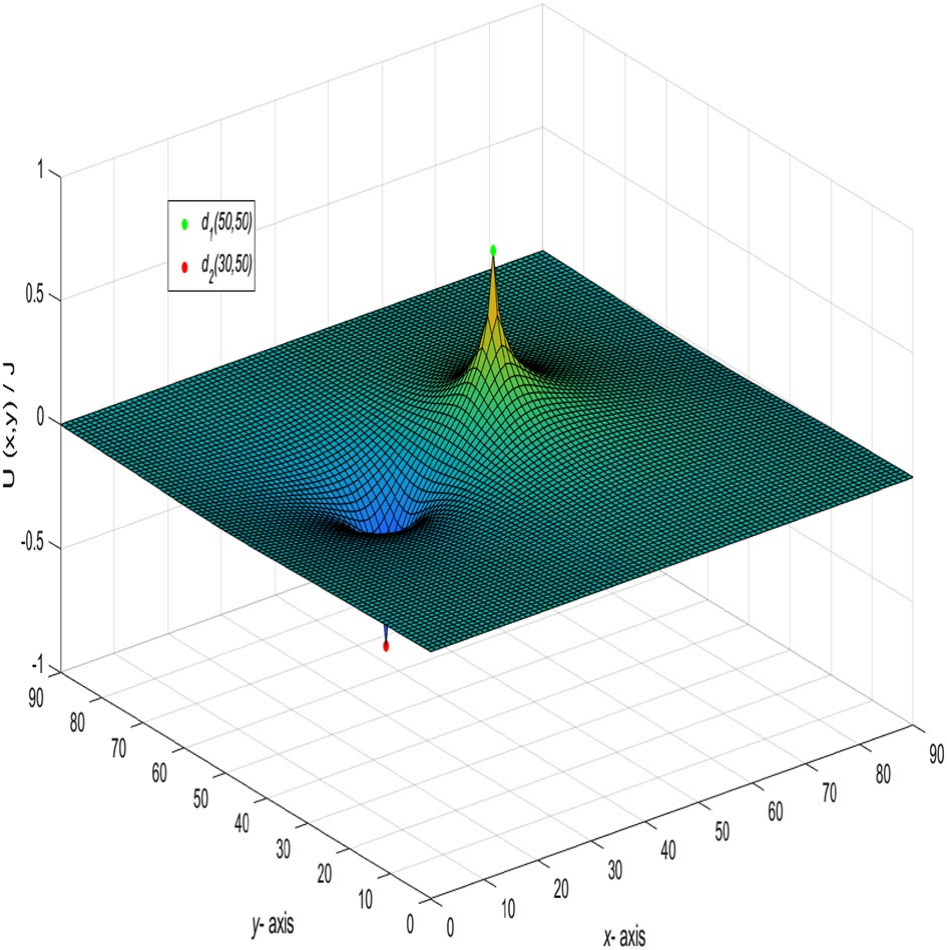
The process of forming a 3D dynamic view for $U_{90\times 90}(x,y)/J$ in Eq. (47).

When $m = n = 90,\ J = 10,\ y_{1} = y_{2} = x_{1} = 50,\ x_{2} = 30,\ and \ r_{0}=r=1,$ the following formula is obtained

47 $$\begin{aligned} \frac{U_{90\times 90}(x,y)}{J} = \frac{1}{45}\sum ^{90}_{j=2}\left( \frac{\gamma ^{(j)}_{50,x} - \gamma ^{(j)}_{30,x}}{t_{j}U^{(j)}_{89}-2U^{(j)}_{88}-2}\right) C_{50,j}C_{y,j}, \end{aligned}$$

where $\gamma ^{(j)}_{50,x}$, $\gamma ^{(j)}_{30,x}$, $C_{50,j}$, $t_{j}$ and $U^{(j)}_{k}$ are the same as Eq. (36), Eq. (40), Eq. (45), Eq. (35) and Eq. (37), respectively, $k=89-|50-x|,~|50-x|-1,89-|30-x|,~|30-x|-1,~89,~88,~~j=1,2,\ldots ,90.$ And a 3D dynamic view is shown in Fig. 8 by Matlab.

**Figure 9:**
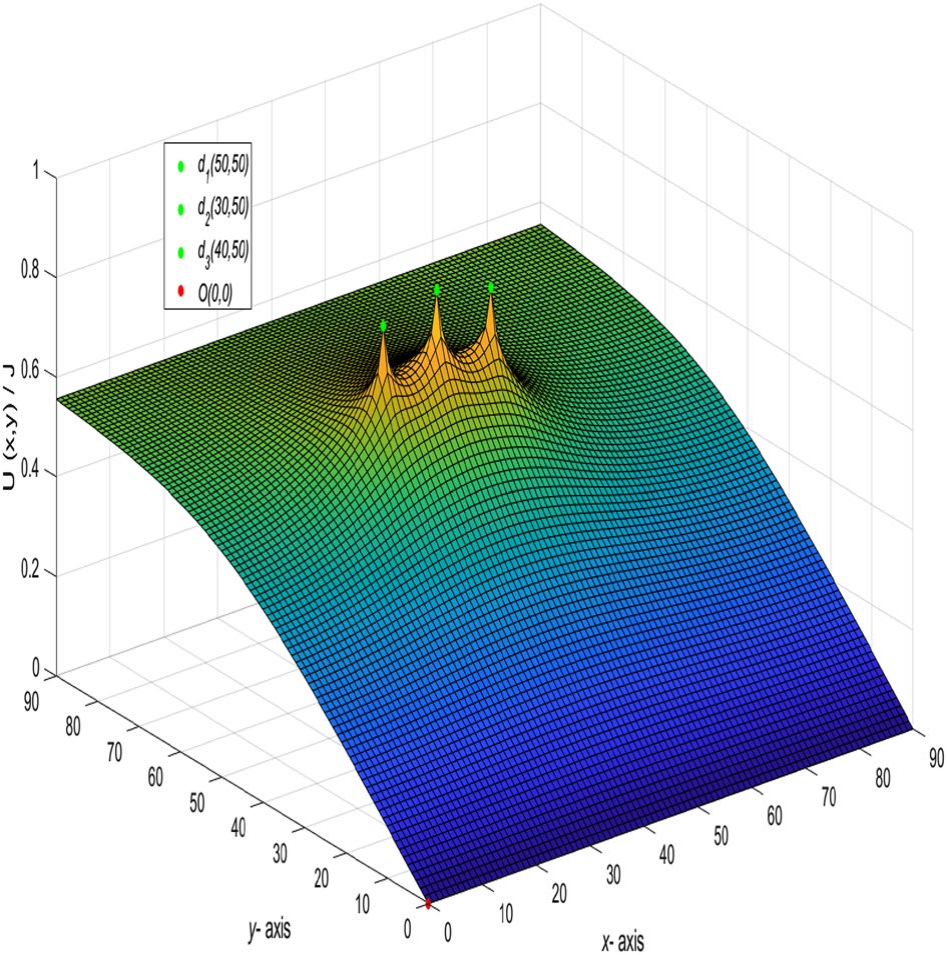
The process of forming a 3D dynamic view for $U_{90\times 90}(x,y)/J$ in Eq. (49).

### Special 6

Presuming that $d_{k}(x_{k}, y_{1}) (k = 1, 2, \ldots k)$ entering the node at the same latitude is *J*/*k* and the current flowing out from *O*(0, 0) is *J*, the potential equation is obtained.

48 $$\begin{aligned} \frac{U_{m\times n}(x,y)}{J} = r_{0}\frac{yy_{1}}{mn} + \frac{2r}{km}\sum ^{m}_{j=2}\frac{C_{y_{1},j}C_{y,j}}{t_{j}U^{(j)}_{n-1}-2U^{(j)}_{n-2}-2}\sum ^{k}_{s=1}\gamma ^{(j)}_{x_{s},x}, \end{aligned}$$

where $C_{k,j}$, $\gamma ^{(j)}_{x_{s},x_{k}}$ and $U^{(j)}_{k}$ are equal to Eq. (7), Eq. (6) and Eq. (9), respectively.

When $m = n = 90,\ J = 10,\ y_{1} = y_{2} = x_{1} = 50,\ x_{2} = 30,\ x_{3} = 40,$ $r_{0}=r=1,\ and \ k=3$ the following formula is obtained

49 $$\begin{aligned} & \quad \frac{U_{90\times 90}(x,y)}{J} = \frac{y}{162}+\frac{1}{45}\sum ^{90}_{j=2}\frac{C_{50,j}C_{y,j}}{t_{j}U^{(j)}_{89}-2U^{(j)}_{88}-2}(\gamma ^{(j)}_{50,x}+\gamma ^{(j)}_{40,x}+\gamma ^{(j)}_{30,x}),\nonumber \\ & \quad \gamma ^{(j)}_{40,x} = U^{(j)}_{89-|40-x|}+U^{(j)}_{|40-x|-1}, \end{aligned}$$

where $C_{50,j}$, $t_{j}$, $\gamma ^{(j)}_{50,x}$, $\gamma ^{(j)}_{30,x}$ and $U^{(j)}_{k}$ is defined in Eq. (45), Eq. (35), Eq. (36), Eq. (40) and Eq. (37)$(k=89-|50-x|,~|50-x|-1,89-|40-x|,~|40-x|-1,89-|30-x|,~|30-x|-1,~89,~88,~~j=1,2,\ldots ,90)$, respectively.

And a 3D dynamic view is shown in Fig. 9 by Matlab.

## Fast numerical algorithm for computing potential

In order to realize fast calculation of potential for large-scale resistor networks. In this section, by summarizing the previous discussion and analysis, a fast numerical algorithm of computing potential by the Eqs. (8), (9), (10), (26), (27), (28), (29), (30), (31), (32) and the famous second type of discrete cosine transform (DCT-II) are given.
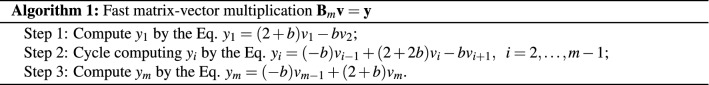

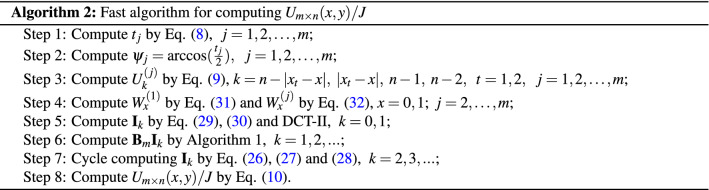


As is well known, the complexity of tridiagonal matrix-vector multiplication is *O*(*n*), which is the same as Algorithm 1. Moreover, one DCT-II needs $2n\log _2 n+ O(n)$ real arithmetic operations^57,58^. So the complexity of Algorithm 2 is $4n\log _2 n+ O(n)$ consist of two DCT-II and Algorithm 1.

According to the above two algorithms, two examples are used to vividly show the computational efficiency for the large scale globe resistor networks.

### Example 1

**Figure 10:**
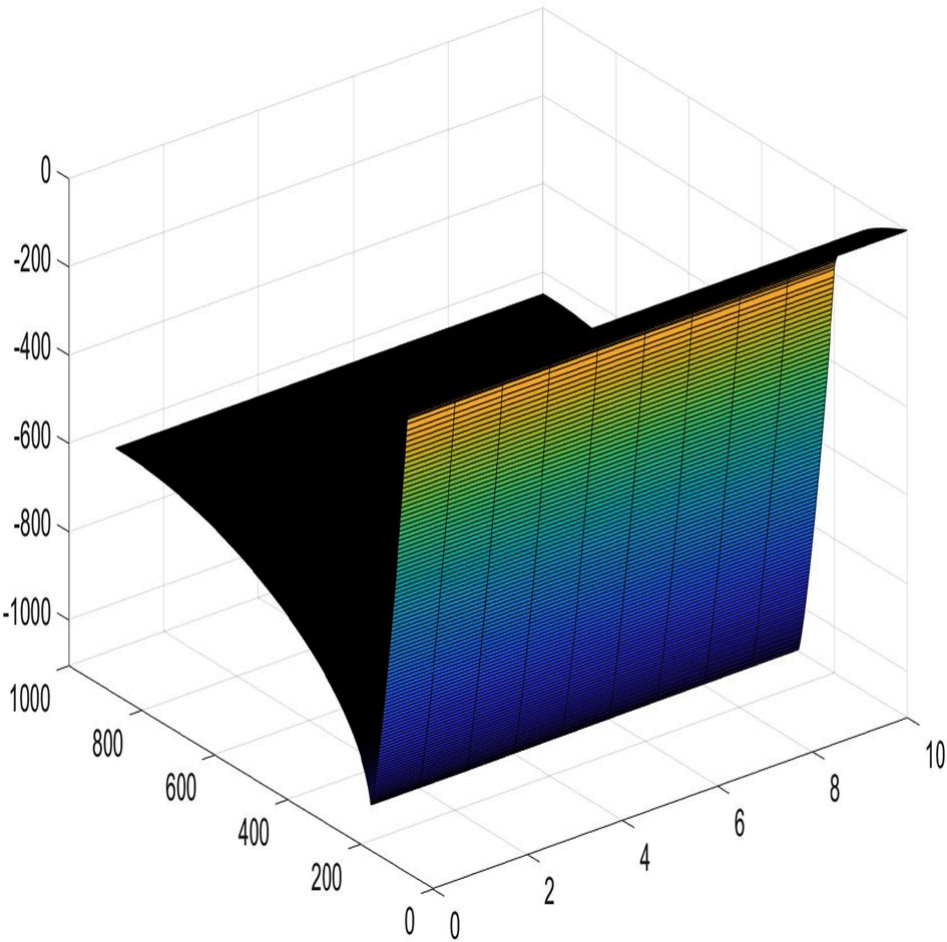
A 3D graph for the changes of $U_{1000\times 10}(x,y)/J$ with *x* and *y*.

In the network with $m = 1000$ and $n = 10$, the current flows in from $(x_{1},y_{1})(x_{1} =3,y_{1} = 200)$ and out from $(x_{2},y_{2})(x_{2} =5,y_{2} = 300)$, $r = 1,~~r_{0} = 100$, and $J = 10$. The results calculated using Algorithm 2 are shown in Fig. 10.

### Example 2

**Figure 11:**
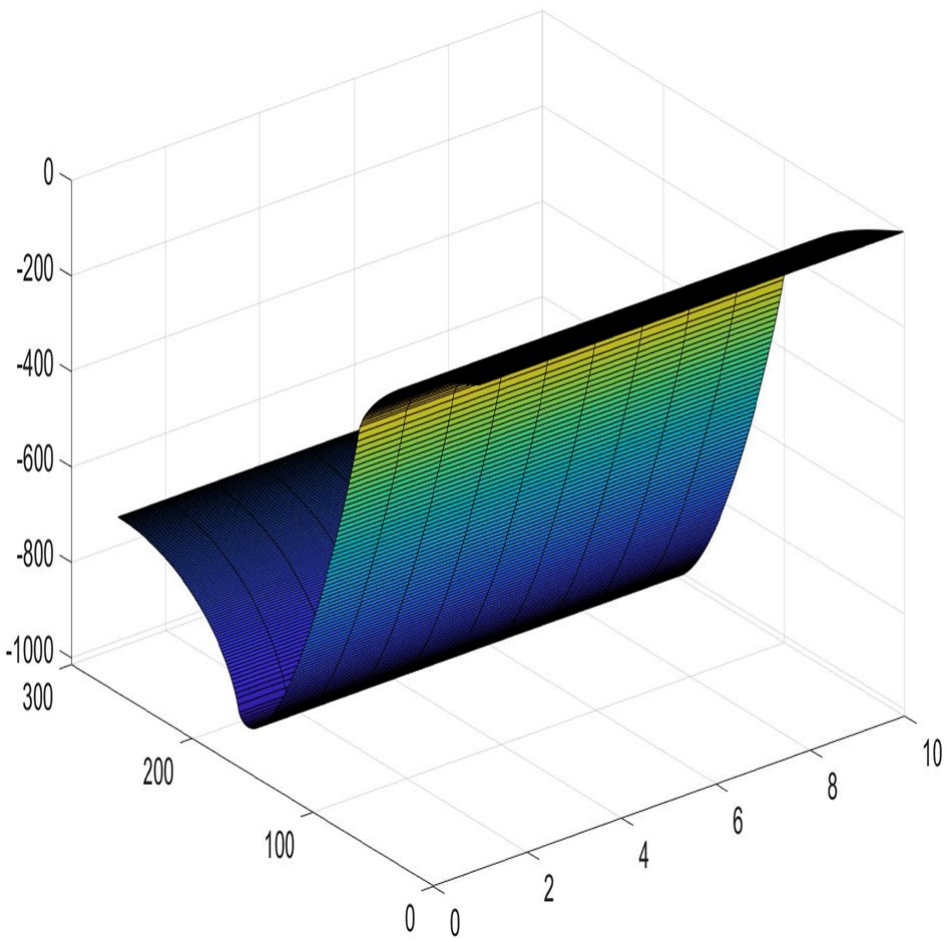
A 3D graph for the changes of $U_{300\times 10}(x,y)/J$ with *x* and *y*.

In the scale of $m \times n (300\times 10)$, when the current $x_{1} =3,\ x_{2} = 5,\ y_{1} =100,\ y_{2} = 200$, and $r=1, r_{0}=100$. The results calculated using Algorithm 2 are shown in Fig. 11.

### Conclusions

This paper achieved a series of improved exact potential formulae in an $m \times n$ globe network by the *RT*-*I* method. Chebyshev polynomial of the second kind is introduced to improve the potential formula of the globe network^44^. Some applications of the new potential formula of the globe network are presented, such as some special and interesting potential formulae are given in Eqs. (33), (38), (41), (43), (46) and (48), respectively. The image numerical simulation using matlab has produced many interesting 3D dynamic views. Finally, we also put forward a fast numerical algorithm by the famous second type of discrete cosine transform, which can realize fast calculation of potential for large-scale resistor networks. Furthermore, by using our research ideas of resistor network, we can also explore neural networks^11–17^. That will be our next step.

## Supplementary Information

Supplementary Information.

## Data Availability

All data generated or analysed during this study are included in this article [and its supplementary information files].
