# Supplementary material for: Fast algorithm and new potential formula represented by Chebyshev polynomials for an m × n globe network
Source: Sci Rep. 2022 Dec 8;12:21260. doi: 10.1038/s41598-022-25724-y (PMC9732363; doi:10.1038/s41598-022-25724-y)
Supplement: Supplementary file 1 — Supplementary Information. [file 41598_2022_25724_MOESM1_ESM.pdf]

Figure 3 matlab code：

```
clc;
clear;
a=90;
b=90;
x1=36;
y1=36;
x2=0;
y2=0;
p=1;
J=10;
r=1;
r0=1;
count_num_single=1;
pic_num=1;
for x1=10 :10 :85
        for y1=10 :10 :85
  x2 = x1 + 10;
  y2 = y1 + 10;
[x,y]= meshgrid(0:1:b-1, 1:1:a);
  L=0;
for i=2:1:a
oi=((i-1).*pi)./a;
ti=2+2.*p-2.*p.*cos(oi);

R=(2.*r./a).*((U(b-abs(x1-x)-1,ti)+U(abs(x1-x)-1,ti)).*sin(y1.*oi)-(U(b-abs(x2-x)-1,ti)+U(abs(x2-x)-1,ti)).*(sin(y2.*oi)))./(ti.*U(b-1,ti)-2.*U(b-2,ti)-2).*(sin(y.*oi));
L=L+R;
end
z=(y.*(y1-y2).*r0./(a.*b))+L;

surf(z)
axis([0 a 0 b -1 1]);
xlabel(' {\it x}- axis');    ylabel(' {\it y}- axis');    zlabel(' U (x,y) / J ');
grid on;
set(gca,'FontSize',16.4)
saveas(gcf,['z' num2str(count_num_single)], 'png');
count_num_single=count_num_single+1;

    %  导出 GIF 格式
                F=getframe(gcf);
                I=frame2im(F);
                [I,map]=rgb2ind(I,200);
```

```
            if pic_num==1
            imwrite(I,map,'one.gif','gif','Loopcount',inf,'DelayTime',1);
            else
               imwrite(I,map,'one.gif','gif','WriteMode','append','DelayTime',1);
            end
            pic_num = pic_num + 1;

    end
end
function[Uki]=U(k,ti)

Uki=(sin(k*acos(ti/2)))/(sin(acos(ti/2)));

end
```

Figure 4 matlab code：

```
clc;
clear;
a=90;
b=90;
x1=50;       % (x1,y1)坐标
y1=50;
x2=0;        % (x2,y2)坐标
y2=0;
r0=1;
J=10;
r=1;

p=1;
I=0;
count_num_single=1;
for f=10:5:90

for x=0:1:b
    for y=0:1:a
 L=0;
for i=2:1:a
oi=((i-1).*pi)./a;
ti=2+2.*p-2.*p.*cos(oi);
R1=(2.*r./a).*((U(b-abs(x1-x)-1,ti)+U(abs(x1-x)-1,ti)).*sin(y1.*oi)-(U(b-abs(x2-x)-1,ti)+U(abs(x2
-x)-1,ti)).*(sin(y2.*oi)))./(ti.*U(b-1,ti)-2.*U(b-2,ti)-2).*(sin(y.*oi));
L=L+R1;
K=L+y*(y1-y2)*r0/(a.*b);
```

```
end
I(y+1,:)=K;
        end
M(:,x+1)=I;
end

Z=M;
z=Z(1:f+1,1:f+1);
[x222,y222]= meshgrid(0:1:f, 0: 1:f);
surf(x222,y222,z);

xlabel(' {\it x}- axis');    ylabel(' {\it y}- axis');    zlabel(' U (x,y) / J ');
hold on;
set(gca,'FontSize',14)
axis([0 a 0 b 0 1.4]);

saveas(gcf,['z' num2str(count_num_single)], 'png');

count_num_single=count_num_single+1;

end
num=count_num_single-1;
for j=1:num
        F=getframe(gcf);
        I=frame2im(F);
%          str = strcat(num2str(j), '.jpg');

        [I,map]=rgb2ind(I,256);
        if(j==1)
                imwrite(I,map,'movefig.gif','gif','LoopCount',inf,'DelayTime',0.1);
        else
                imwrite(I,map,'movefig.gif','gif','WriteMode','append','DelayTime',0.1);
        end
end
function[Uki]=U(k,ti)

Uki=(sin((k+1)*acos(ti/2)))/(sin(acos(ti/2)));

end
```

Figure 5 matlab code：

```
clc;
clear;
```

```
a=90;
b=90;
x1=0;          % (x1,y1)坐标
y1=0;
x2=30;          % (x2,y2)坐标
y2=30;
r0=1;
J=10;
r=1;

p=1;
I=0;
count_num_single=1;
for f=10:5:90

for x=0:1:b
        for y=0:1:a
  L=0;
for i=2:1:a
oi=((i-1).*pi)./a;
ti=2+2.*p-2.*p.*cos(oi);
R1=(2.*r./a).*((U(b-abs(x1-x)-1,ti)+U(abs(x1-x)-1,ti)).*sin(y1.*oi)-(U(b-abs(x2-x)-1,ti)+U(abs(x2
-x)-1,ti)).*(sin(y2.*oi)))./(ti.*U(b-1,ti)-2.*U(b-2,ti)-2).*(sin(y.*oi));
L=L+R1;
K=L+y*(y1-y2)*r0/(a.*b);
end
I(y+1,:)=K;
        end
M(:,x+1)=I;
end

Z=M;
z=Z(1:f+1,1:f+1);
[x222,y222]= meshgrid(0:1:f, 0: 1:f);
surf(x222,y222,z);

xlabel(' {\it x}- axis');    ylabel(' {\it y}- axis');    zlabel(' U (x,y) / J ');
hold on;
set(gca,'FontSize',14)
axis([0 a 0 b -1.5 1]);

saveas(gcf,['z' num2str(count_num_single)], 'png');

count_num_single=count_num_single+1;
```

```
end
num=count_num_single-1;
for j=1:num
    F=getframe(gcf);
    I=frame2im(F);
%       str = strcat(num2str(j), '.jpg');

    [I,map]=rgb2ind(I,256);
    if(j==1)
        imwrite(I,map,'movefig.gif','gif','LoopCount',inf,'DelayTime',0.1);
    else
        imwrite(I,map,'movefig.gif','gif','WriteMode','append','DelayTime',0.1);
    end
end
function[Uki]=U(k,ti)

Uki=(sin((k+1)*acos(ti/2)))/(sin(acos(ti/2)));

end
```

Figure 6 matlab code：

```
clc;
clear;
a=290;
b=290;
x1=0;       % (x1,y1)坐标
y1=30;
x2=30;      % (x2,y2)坐标
y2=0;
r0=1;
J=10;
r=1;

p=1;
I=0;
count_num_single=1;

for f=10:5:90

for x=0:1:b
    for y=0:1:a
  L=0;
```

```
for i=2:1:a
oi=((i-1).*pi)./a;
ti=2+2.*p-2.*p.*cos(oi);
R1=(2.*r./a).*((U(b-abs(x1-x)-1,ti)+U(abs(x1-x)-1,ti)).*sin(y1.*oi)-(U(b-abs(x2-x)-1,ti)+U(abs(x2
-x)-1,ti)).*(sin(y2.*oi)))./(ti.*U(b-1,ti)-2.*U(b-2,ti)-2).*(sin(y.*oi));
L=L+R1;
K=L+y*(y1-y2)*r0/(a.*b);
end
I(y+1,:)=K;
    end
M(:,x+1)=I;
end

Z=M;
z=Z(1:f+1,1:f+1);
[x222,y222]= meshgrid(0:1:f, 0: 1:f);
surf(x222,y222,z);

xlabel(' {\it x}- axis');    ylabel(' {\it y}- axis');    zlabel(' U (x,y) / J ');
hold on;
set(gca,'FontSize',14)
axis([0 a 0 b -1.2 1.2]);

saveas(gcf,['z' num2str(count_num_single)], 'png');

count_num_single=count_num_single+1;

end
num=count_num_single-1;
for j=1:num
    F=getframe(gcf);
    I=frame2im(F);
%       str = strcat(num2str(j), '.jpg');

    [I,map]=rgb2ind(I,256);
    if(j==1)
        imwrite(I,map,'movefig.gif','gif','LoopCount',inf,'DelayTime',0.1);
    else
        imwrite(I,map,'movefig.gif','gif','WriteMode','append','DelayTime',0.1);
    end
end
function[Uki]=U(k,ti)
Uki=(sin((k+1)*acos(ti/2)))/(sin(acos(ti/2)));
end
```

Figure 7 matlab code：

```
clc;
clear;
a=90;
b=90;
x1=50;
y1=50;
x2=50;
y2=30;
r0=1;
J=10;
r=1;

p=1;
I=0;
count_num_single=1;

for f=10:5:90

for x=0:1:b
        for y=0:1:a
  L=0;
for i=2:1:a
oi=((i-1).*pi)./a;
ti=2+2.*p-2.*p.*cos(oi);
R1=(2.*r./a).*((U(b-abs(x1-x)-1,ti)+U(abs(x1-x)-1,ti)).*sin(y1.*oi)-(U(b-abs(x2-x)-1,ti)+U(abs(x2
-x)-1,ti)).*(sin(y2.*oi)))./(ti.*U(b-1,ti)-2.*U(b-2,ti)-2).*(sin(y.*oi));
L=L+R1;
K=L+y*(y1-y2)*r0/(a.*b);
end
I(y+1,:)=K;
        end
M(:,x+1)=I;
end

Z=M;
z=Z(1:f+1,1:f+1);
[x222,y222]= meshgrid(0:1:f, 0: 1:f);
surf(x222,y222,z);

xlabel(' {\it x}- axis');    ylabel(' {\it y}- axis');    zlabel(' U (x,y) / J ');
hold on;
set(gca,'FontSize',14)
```

```
axis([0 a 0 b -1 1]);

saveas(gcf,['z' num2str(count_num_single)], 'png');

count_num_single=count_num_single+1;

end
num=count_num_single-1;
for j=1:num
    F=getframe(gcf);
    I=frame2im(F);
%       str = strcat(num2str(j), '.jpg');

    [I,map]=rgb2ind(I,256);
    if(j==1)
        imwrite(I,map,'movefig.gif','gif','LoopCount',inf,'DelayTime',0.1);
    else
        imwrite(I,map,'movefig.gif','gif','WriteMode','append','DelayTime',0.1);
    end
end
function[Uki]=U(k,ti)

Uki=(sin((k+1)*acos(ti/2)))/(sin(acos(ti/2)));

end
```

Figure 8 matlab code：

```
clc;
clear;
a=90;
b=90;
x1=50;      % (x1,y1)坐标
y1=50;
x2=30;      % (x2,y2)坐标
y2=50;
r0=1;
J=10;
r=1;

p=1;
I=0;
count_num_single=1;
for f=10:5:90
```

```
for x=0:1:b
        for y=0:1:a
  L=0;
for i=2:1:a
oi=((i-1).*pi)./a;
ti=2+2.*p-2.*p.*cos(oi);
R1=(2.*r./a).*((U(b-abs(x1-x)-1,ti)+U(abs(x1-x)-1,ti)).*sin(y1.*oi)-(U(b-abs(x2-x)-1,ti)+U(abs(x2
-x)-1,ti)).*(sin(y2.*oi)))./(ti.*U(b-1,ti)-2.*U(b-2,ti)-2).*(sin(y.*oi));
L=L+R1;
K=L+y*(y1-y2)*r0/(a.*b);
end
I(y+1,:)=K;
        end
M(:,x+1)=I;
end

Z=M;
z=Z(1:f+1,1:f+1);
[x222,y222]= meshgrid(0:1:f, 0: 1:f);
surf(x222,y222,z);

xlabel(' {\it x}- axis');    ylabel(' {\it y}- axis');    zlabel(' U (x,y) / J ');
hold on;
set(gca,'FontSize',14)
axis([0 a 0 b -1 1]);

saveas(gcf,['z' num2str(count_num_single)], 'png');

count_num_single=count_num_single+1;

end
num=count_num_single-1;
for j=1:num
        F=getframe(gcf);
        I=frame2im(F);
%          str = strcat(num2str(j), '.jpg');

        [I,map]=rgb2ind(I,256);
        if(j==1)
                imwrite(I,map,'movefig.gif','gif','LoopCount',inf,'DelayTime',0.1);
        else
                imwrite(I,map,'movefig.gif','gif','WriteMode','append','DelayTime',0.1);
        end
```

```
end
function[Uki]=U(k,ti)

Uki=(sin((k+1)*acos(ti/2)))/(sin(acos(ti/2)));

end
```

Figure 9 matlab code：

```
clc;
clear;
a=90;
b=90;
J=10;
x1=50;
y1=50;
x2=30;
y2=50;

p=1;
I=0;
r=1;
r0=1;
L=0;
c=3;
x3=40;

count_num_single=1;
for f=10:5:90

for x=0:1:b
       for y=0:1:a
  L=0;
for i=2:1:a
oi=((i-1).*pi)./a;
ti=2+2.*p-2.*p.*cos(oi);
R1=(2*r/(a*c)*sin(y1*oi)*sin(y.*oi)/(ti*U(b-1,ti)-2*U(b-2,ti)-2))*((U(b-abs(x1-x)-1,ti)+U(abs(x1
-x)-1,ti))+(U(b-abs(x2-x)-1,ti)+U(abs(x2-x)-1,ti))+(U(b-abs(x3-x)-1,ti)+U(abs(x3-x)-1,ti)));
L=L+R1;
K=L+(r0*y*y1/a/b);
end
I(y+1,:)=K;
       end
M(:,x+1)=I;
```

```
end
Z=M;
z=Z(1:f+1,1:f+1);
[x222,y222]= meshgrid(0:1:f, 0: 1:f);
surf(x222,y222,z);
axis([0 a 0 b 0 1]);

xlabel(' {\it x}- axis');    ylabel(' {\it y}- axis');    zlabel(' U (x,y) / J ');
grid on;
set(gca,'FontSize',14)
saveas(gcf,['z' num2str(count_num_single)], 'png');
count_num_single=count_num_single+1;
end
num=count_num_single-1;
for j=1:num
    F=getframe(gcf);
    I=frame2im(F);
%       str = strcat(num2str(j), '.jpg');

    [I,map]=rgb2ind(I,256);
    if(j==1)
        imwrite(I,map,'movefig.gif','gif','LoopCount',inf,'DelayTime',0.1);
    else
        imwrite(I,map,'movefig.gif','gif','WriteMode','append','DelayTime',0.1);
    end
end

function[Uki]=U(k,ti)

Uki=(sin((k+1)*acos(ti/2)))/(sin(acos(ti/2)));

end
```

Figure 10 matlab code：

```
clc;
clear;
a=1000;
b=10;
x1=3;       % (x1,y1)坐标
y1=200;
x2=5;       % (x2,y2)坐标
y2=300;
r0=100;
J=10;
```

```
r=1;
rho=0.01;       % rho=r/r0
L=0;
M=0;

for i=2:1:a
        oi=(i-1)*pi/a;
        ti=2+2*rho-2*rho*cos(oi);
        delta1=-2*sin(0.5*oi)*sin(y1*oi);
        delta2=2*sin(0.5*oi)*sin(y2*oi);

W0(i,:)=rho*J*((V_func(b-abs(x1-0)-1,ti)+V_func(abs(x1-0)-1,ti))*delta1+(V_func(b-abs(x2-0)-1,
ti)+V_func(abs(x2-0)-1,ti))*delta2)./(ti.*V_func(b-1,ti)-2.*V_func(b-2,ti)-2);
W1(i,:)=rho*J*((V_func(b-abs(x1-1)-1,ti)+V_func(abs(x1-1)-1,ti))*delta1+(V_func(b-abs(x2-1)-1,
ti)+V_func(abs(x2-1)-1,ti))*delta2)./(ti.*V_func(b-1,ti)-2.*V_func(b-2,ti)-2);
W0(1,:)=J*(y2-y1)./(b.*sqrt(2));
W1(1,:)=J*(y2-y1)./(b.*sqrt(2));

end

I(:,1)=sqrt(2/a)*dct(W0);
I(:,2)=sqrt(2/a)*dct(W1);
for i=3:1:b
          H=zeros(a,1);
        if isequal(i-1,x1)
                H(y1)=-1;H(y1+1)=1;
                I(:,i)=PI_func(rho,I(:,i-1))-I(:,i-2)-rho*J*H;
        elseif isequal(i-1,x2)
                H(y2)=1;H(y2+1)=-1;
                I(:,i)=PI_func(rho,I(:,i-1))-I(:,i-2)-rho*J*H;
        else
                I(:,i)=PI_func(rho,I(:,i-1))-I(:,i-2);
        end

end
for x=1:1:b
        for y=1:1:a
sigma(y,x)=sum(I(1:y,x));
output=-r0*(sigma)/J;
        end
end
z=output;
mesh(z)
xlabel(' {\it x}- axis');    ylabel(' {\it{h}}- axis');    zlabel(' I ');
```

```
figure;
surf(z)
set(gca,'FontSize',20)

function y=PI_func(rho,I)
%-----------------算法 1：计算三对角矩阵 B 和向量 I 的乘积-------------------%

a=length(I);
y = zeros(a,1);
y(1) = (2+rho)*I(1) - rho*I(2); % 第一个值
for i = 2:a-1
    y(i) = (-rho)*I(i-1)+(2+2*rho)*I(i)-rho*I(i+1); % 循环求解 y 的第 2 个值到第 a-1 个值
end
y(a) = (-rho)*I(a-1)+(2+rho)*I(a); % 最后一个值
function Uki=V_func(k,ti)
Uki=(sin((k+1)*acos(ti/2)))/(sin(acos(ti/2)));
```

Figure 11 matlab code：

```
clc;
clear;
a=300;
b=10;
x1=3;        % (x1,y1)坐标
y1=100;
x2=5;        % (x2,y2)坐标
y2=200;
r0=100;
J=10;
r=1;
rho=0.01;      % rho=r/r0
L=0;
M=0;

for i=2:1:a
    oi=(i-1)*pi/a;
    ti=2+2*rho-2*rho*cos(oi);
    delta1=-2*sin(0.5*oi)*sin(y1*oi);
    delta2=2*sin(0.5*oi)*sin(y2*oi);

W0(i,:)=rho*J*((V_func(b-abs(x1-0)-1,ti)+V_func(abs(x1-0)-1,ti))*delta1+(V_func(b-abs(x2-0)-1,
ti)+V_func(abs(x2-0)-1,ti))*delta2)./(ti.*V_func(b-1,ti)-2.*V_func(b-2,ti)-2);
W1(i,:)=rho*J*((V_func(b-abs(x1-1)-1,ti)+V_func(abs(x1-1)-1,ti))*delta1+(V_func(b-abs(x2-1)-1,
ti)+V_func(abs(x2-1)-1,ti))*delta2)./(ti.*V_func(b-1,ti)-2.*V_func(b-2,ti)-2);
```

```
W0(1,:)=J*(y2-y1)./(b.*sqrt(2));
W1(1,:)=J*(y2-y1)./(b.*sqrt(2));

end
I(:,1)=sqrt(2/a)*dct(W0);
I(:,2)=sqrt(2/a)*dct(W1);
for i=3:1:b
     H=zeros(a,1);
    if isequal(i-1,x1)
        H(y1)=-1;H(y1+1)=1;
        I(:,i)=PI_func(rho,I(:,i-1))-I(:,i-2)-rho*J*H;
    elseif isequal(i-1,x2)
        H(y2)=1;H(y2+1)=-1;
        I(:,i)=PI_func(rho,I(:,i-1))-I(:,i-2)-rho*J*H;
    else
        I(:,i)=PI_func(rho,I(:,i-1))-I(:,i-2);
    end

end
for x=1:1:b
    for y=1:1:a
sigma(y,x)=sum(I(1:y,x));
output=-r0*(sigma)/J;
    end
end
z=output;
mesh(z)
xlabel(' {\it x}- axis');    ylabel(' {\it{h}}- axis');    zlabel(' I ');
figure;
surf(z)
set(gca,'FontSize',20)
function y=PI_func(rho,I)
%------------------算法 1：计算三对角矩阵 B 和向量 I 的乘积--------------------%
a=length(I);
y = zeros(a,1);
y(1) = (2+rho)*I(1) - rho*I(2); %  第一个值
for i = 2:a-1
    y(i) = (-rho)*I(i-1)+(2+2*rho)*I(i)-rho*I(i+1); %  循环求解 y 的第 2 个值到第 a-1 个值
end
y(a) = (-rho)*I(a-1)+(2+rho)*I(a); %  最后一个值
function Uki=V_func(k,ti)
Uki=(sin((k+1)*acos(ti/2)))/(sin(acos(ti/2)));
```
